# Effects of Conservative Oxygen Therapy versus Conventional Oxygen Therapy on the Mortality in ICU Patients: A Meta-Analysis

**DOI:** 10.1155/2023/7023712

**Published:** 2023-10-14

**Authors:** Xinyu Jiang, Dong Qiu

**Affiliations:** The First Affiliated Hospital of Soochow University, Suzhou 215006, China

## Abstract

**Objective:**

To compare the effects of conservative oxygen therapy and conventional oxygen therapy on the mortality of critically ill patients in ICU.

**Methods:**

Searching for randomized controlled clinical trials (RCT) on the effect of conservative oxygen therapy and conventional oxygen therapy on the mortality of critically ill patients in computer databases, including PubMed, Embase, Cochrane Library, CNKI, VIP, and Wanfang, with postdate before August 2022. We have two researchers evaluating the quality of the literature included and extracting data as per the inclusion and exclusion criteria and then analyzed it with RevMan 5.4 statistical software. Primary outcome included short-term mortality (28-day mortality or ICU mortality); secondary outcome included 90-day mortality, ICU length of stay, hospital length of stay, incidence of new organ dysfunction in ICU, incidence of new infection in ICU, and incidence of ICUAW.

**Results:**

A total of 5779 subjects were included in 10 articles, including 2886 in the conservative oxygen therapy group and 2893 in the conventional oxygen therapy group. The meta-analysis showed that conservative oxygen therapy had an advantage over conventional oxygen therapy in terms of short-term mortality (*P*=0.03). Subgroup analysis based on different conservative oxygen targets showed that this advantage was statistically significant when the target is set above 90% (RR = 0.76, 95% CI = 0.62∼0.94, *P*=0.01), while there was no significant difference between conservative oxygen therapy and conventional oxygen therapy when the target is set below 90% (RR = 0.95, 95% CI = 0.79∼1.16, *P*=0.63). In addition, in terms of the incidence of new infections in the ICU (*P*=0.03) and the incidence of ICUAW (*P*=0.03), conservative oxygen therapy also had advantages over conventional oxygen therapy, and the difference was statistically significant. But in terms of 90-day mortality (*P*=0.61), ICU length of stay (*P*=0.96), hospital length of stay (*P*=0.47), and incidence of new organ dysfunction in ICU (*P*=0.61), there was no significant difference between conservative oxygen therapy and conventional oxygen therapy.

**Conclusion:**

Compared with conventional oxygen therapy, conservative oxygen therapy can reduce the short-term mortality of severe patients, especially when the conservative oxygen therapy target is set above 90%. And it can also reduce the incidence of ICU new infections and ICUAW, while having no effect on 90-day mortality, ICU length of stay, and hospital length of stay.

## 1. Introduction

Oxygen therapy is a widely used treatment method in critically ill patients. Due to the patients' inspired oxygen concentration (FiO_2_) often exceeding ambient oxygen concentration during ICU stay, they often reach an excessive arterial partial pressure of oxygen (PaO_2_) level within 24 hours of admission [1, 2]. In this case, the hyperoxia can compensate and prevent tissue hypoxia by promoting oxygen delivery to affected organs [3]. However, studies have shown that prolonged exposure to hyperoxemia can also be harmful. Hyperoxia may result in acute lung injury, atelectasis, or increased risk of infection due to oxidative stress and inflammation. In addition, hyperoxia can result in vasoconstriction, reducing coronary blood flow and cardiac output, and alter microvascular perfusion [4, 5].

In an observational study from the Netherlands, the in-hospital mortality was found to be linearly associated with FiO_2_ among ICU patients and U-shaped with PaO_2_ (i.e., lower and higher PaO_2_ were both associated with higher mortality), with both independent of each other [6]. Therefore, the concept of conservative oxygen therapy strategy has been proposed. However, the opinions on the setting of oxygen therapy standards and optimal oxygenation goals are still inconsistent in various clinical guidelines [7–9].

In a randomized clinical trial of optimal oxygenation in ICU patients published in 2016, patients receiving oxygen therapy according to a conservative strategy (PaO_2_ of 70–100 mmHg or arterial oxygen saturation (SpO_2_) of 94–98%) have an improved ICU mortality compared with the conventional control group (PaO_2_ up to 150 mmHg or SpO_2_ 97–100%) [10]. This trial is the first RCT to demonstrate the potential harm of conventional oxygen therapy. Some earlier observational studies also proved this point [11–13].

However, some similar randomized controlled studies afterwards found that conservative oxygen therapy did not improve patient survival [14, 15]. Therefore, uncertainty remains about optimal oxygenation goals for ICU patients. To address the limitations of previous analyses, we attempted to perform a meta-analysis by searching existing RCT studies to compare the impact of these two oxygen therapy strategies on mortality in critically ill patients.

## 2. Methods

### 2.1. Inclusion and Exclusion Criteria

#### 2.1.1. Types of Studies

The types of studies included the published RCT studies at home and abroad on the effect of conservative oxygen therapy and conventional oxygen therapy on the mortality of critically ill patients. Language is limited to Chinese and English.

#### 2.1.2. Research Objects

Research objects included ICU patients aged ≥18 years.

#### 2.1.3. Interventions

Inventions included conservative oxygen therapy and conventional oxygen therapy.

#### 2.1.4. Outcome Indicators

Primary outcome indicators included short-term mortality rate (28-day fatality rate or ICU fatality rate). Secondary outcome indicators included 90-day fatality rate, ICU length of stay, hospital length of stay, incidence of new organ dysfunction in ICU, incidence of new infections in ICU, and ICUAW incidence.

#### 2.1.5. Exclusion Metrics

Exclusion metrics included the following: ① conference papers and abstracts; ② data cannot be extracted; and ③ repeated research.

### 2.2. Literature Search Strategy

Databases were searched (PubMed, Embase, Cochrane Library, CNKI, VIP, and Wanfang) and RCT studies were collected that compared effects of conservative oxygen therapy and conventional oxygen therapy on the mortality of critically ill patients, with postdate before August 2022. The search uses a combination of subject headings and free words and traces the references included in the literature to supplement the acquisition of the relevant literature. Chinese search terms include “氧疗,” “病死率,” and “重症患者”; English search terms include conservative oxygen, liberal oxygen, conventional oxygen, hypoxia, hyperoxia, oxygen deficiencies, hypoxemia, anoxia, critical Illness, critical Care, intensive care units, and randomized controlled trial.

### 2.3. Literature Screening and Data Extraction

Two researchers independently screened the literature, extracted data, and cross-checked. If there were any differences, they were resolved through discussion. For the literature lacking information, try to get in touch with the original author to supplement it. The extracted data included ① basic information of included studies, including author's name and publication year; ② basic characteristics of research subjects, including sample size and patient type; ③ intervention measures including SpO_2_ and PaO_2_ levels of conservative oxygen therapy and conventional oxygen therapy; ④ key elements of risk of bias assessment; and ⑤ main data of outcome indicators concerned.

### 2.4. Risk of Bias Assessment of Included Studies

The risk of bias assessment of the included studies was assessed using the risk of bias assessment tool for RCTs recommended by the Cochrane Handbook version 5.1.0: ① whether the randomization method was correct; ② whether the allocation was concealed; ③ whether subjects and investigators were blinded; ④ completeness of outcome data; ⑤ whether the results of the study were selectively reported; and ⑥ other sources of bias. The risk of bias was assessed independently by 2 reviewers, and the results were cross-checked. In case of disagreements, they were resolved through discussion.

### 2.5. Statistical Analysis

RevMan 5.4 statistical software was used for meta-analysis. The relative risk (RR) was used for enumeration data, and the standardized mean difference (SMD) was used for measurement data as efficacy analysis statistics. *P* < 0.05 was considered to be statistically significant. The heterogeneity of the included studies was analyzed by the *X*^2^ test (the test level was *α* = 0.1), and the *I*^2^ statistic was used for evaluation. If the heterogeneity test result *I*^2^ < 50%, a fixed-effects model is used for meta-analysis; if the heterogeneity test result *I*^2^ ≥ 50%, it indicates that there is statistical heterogeneity among the results of each study, thus further analysis of heterogeneity is required, and meta-analysis was performed using a random-effects model after excluding significant clinical and methodological heterogeneity. Publication bias was assessed by drawing a funnel plot.

## 3. Results

### 3.1. Search Result

**Figure 1:**
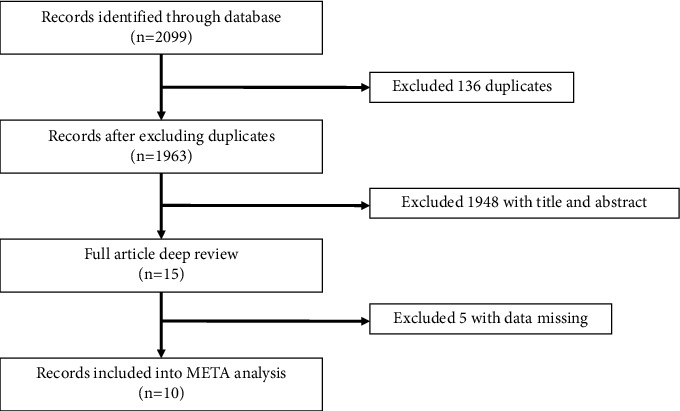
Literature screening process and result.

A total of 2099 related literature studies were retrieved. After reading the literature titles and abstracts, according to the inclusion and exclusion criteria, 10 RCT studies with a total of 5779 patients were finally included. The screening process is shown in Figure 1, and the basic characteristics of the included studies are shown in Table 1.

### 3.2. Quality Evaluation of Included Literature

The 10 included studies were all RCT studies, of which 7 were randomly generated by computer randomization scheme and 3 were generated by a random list. 9 articles described allocation concealment, 2 were double-blind, and another 2 were single-blind. The results of literature quality evaluation are shown in Sup 1.

### 3.3. Meta-Analysis Results

#### 3.3.1. Effects on Short-Term Mortality

**Figure 2:**
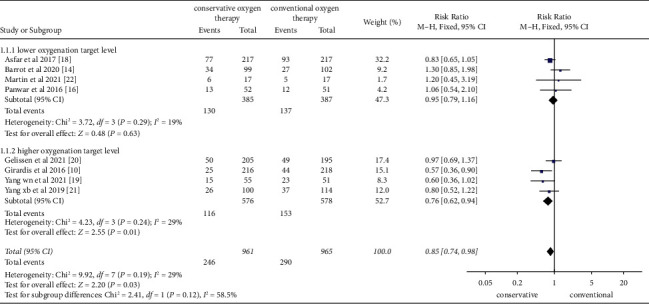
Meta-analysis of short-term mortality in different groups.

Five studies [10, 16, 19, 20, 22] described ICU mortality and three studies [14, 18, 21] described 28-day mortality, with no heterogeneity (*P*=0.19, *I*^2^ = 29%) among studies. Therefore, a fixed-effects model was used for meta-analysis. Results showed that conservative oxygen therapy had an advantage over conventional oxygen therapy in terms of short-term mortality (RR = 0.85, 95% CI = 0.74–0.98, *P*=0.03). Subgroup analysis based on different conservative oxygen targets showed that this advantage was statistically significant when the target was set above 90% (RR = 0.76, 95% CI = 0.62∼0.94, *P*=0.01), while there was no significant difference between conservative oxygen therapy and conventional oxygen therapy when the target is set below 90% (RR = 0.95, 95% CI = 0.79∼1.16, *P*=0.63). Results are shown in Figure 2.

#### 3.3.2. Effects on 90-Day Mortality Rate

Six studies [14–18, 22] provided the 90-day mortality rate data, with no heterogeneity among the studies (*P*=0.26, *I*^2^ = 24%). Therefore, a fixed-effects model was used for meta-analysis. The results showed that there was no statistical significance regarding the difference in 90-day mortality between conservative oxygen therapy and conventional oxygen therapy (RR = 1.02, 95% CI = 0.95–1.09, *P*=0.61). The results are shown in Sup 2.

#### 3.3.3. Effects on ICU Length of Stay

Five studies [10, 16, 18–20] described ICU length of stay, with no heterogeneity among studies (*P*=0.20, *I*^2^ = 33%), and thus, a fixed-effects model was used for meta-analysis. The results showed that there was no statistical significance regarding the difference in ICU length of stay between conservative oxygen therapy and conventional oxygen therapy groups (SMD = −0.02, 95% CI = −0.12–0.08, *P*=0.72). The results are shown in Sup 3.

#### 3.3.4. Effects on Hospital Length of Stay

Two studies [10, 16] described the hospital length of stay, and there was no heterogeneity among the studies (*P*=0.21, *I*^2^ = 37%), and thus, a fixed-effects model was used for meta-analysis. The results showed that there was no statistical significance regarding the difference in hospital length of stay between conservative oxygen therapy and conventional oxygen therapy groups (SMD = 0.05, 95% CI = −0.12–0.22, *P*=0.54). The results are shown in Sup 4.

#### 3.3.5. Effect on Incidence of New ICU Organ Dysfunction

Six studies [10, 14, 17–20] provided data on incidence of new ICU organ dysfunction, including myocardial infarction, shock, liver and kidney failure, and intestinal ischemia. Heterogeneity was found among the studies (*P*=0.005, *I*^2^ = 70%), and thus, a random-effects model was used for meta-analysis. The results showed that there was no statistical significance regarding the difference in incidence of new ICU organ dysfunction between conservative oxygen therapy and conventional oxygen (RR = 0.96, 95% CI = 0.83–1.12, *P*=0.61). The results are shown in Sup 5.

#### 3.3.6. Effect on Incidence of New ICU Infections

Four studies [10, 14, 18, 19] provided data on ICU new infections, including lung infections, bloodstream infections, and urinary tract infections, with no heterogeneity among studies (*P*=0.45, *I*^2^ = 0%), and thus, a fixed-effects model was used for meta-analysis. The results showed that conservative oxygen therapy had an advantage over conventional oxygen therapy in terms of the incidence of new ICU infections, and the difference was statistically significant (RR = 0.8, 95% CI = 0.66–0.98, *P*=0.03). The results are shown in Sup 6.

#### 3.3.7. Effect on Incidence of ICUAW

Two studies [18, 19] described ICUAW, and there was no heterogeneity between studies (*P*=0.84, *I*^2^ = 0%); therefore, a fixed-effects model was used for meta-analysis. The results showed that conservative oxygen therapy had an advantage over conventional oxygen therapy in terms of the incidence of ICUAW, and the difference was statistically significant (RR = 0.53, 95% CI = 0.29–0.94, *P*=0.03). The results are shown in Sup 7.

#### 3.3.8. Publication Bias Results

The funnel plots of studies on short-term mortality in the included literature were asymmetric, indicating publication bias. The results are shown in Sup 8.

## 4. Discussion

This study is a meta-analysis of the effect of conservative oxygen therapy and conventional oxygen therapy on the mortality of critically ill patients. The results show that the conservative oxygen therapy can reduce the short-term mortality rate of critically ill patients compared with conventional oxygen therapy. The advantage of conservative oxygen therapy over conventional oxygen therapy in terms of short-term mortality is statistically significant, especially when the conservative oxygen therapy target is set above 90%. In addition, the conservative oxygen therapy can also reduce the incidence of new ICU infections as well as the incidence of ICUAW, but there was no statistical significance regarding the difference in 90-day mortality, ICU length of stay, hospital length of stay, and incidence of new ICU organ dysfunction between the two groups.

Our findings suggest an association between hyperoxemia and increased mortality in critically ill patients, consistent with the findings of an observational study published in 2017, which found that patients with PaO_2_ between 120 and 200 mmHg had lower mortality than patients with PaO_2_ ≥ 200 mmHg, and the duration of hyperoxemia was positively correlated with in-hospital mortality [23]. Now, a number of clinical studies have confirmed that hyperoxia can cause damage to the body, such as atelectasis and pulmonary interstitial fibrosis, increase the risk of lower respiratory tract infection, leading to lung damage [24], or cause coronary artery contraction, excite the vagus nerve, reduce cardiac output and myocardial blood supply, leading to myocardial damage [25], or induce apoptosis of normal brain tissue cells, causing repeated cerebral ischemia, resulting in brain tissue damage [26]. Two recently published meta-analyses [2, 27] showed that compared with open and conservative oxygen therapy, conservative oxygen therapy strategies can reduce mortality, which is consistent with the results of this meta-analysis.

In this meta-analysis, conservative oxygen therapy can improve short-term mortality in critically ill patients. However, comparing the 90-day mortality rate, the difference between the two was not statistically significant. This may be related to the fact that only one study included in this outcome indicator excluded patients with severe hypoxic respiratory failure. Our findings do not support the use of conservative oxygen therapy in ICU patients with severe hypoxic respiratory failure. Theoretically, these patients have more severe gas exchange disturbances and refractory hypoxemia, requiring more intensive respiratory support [28]. In addition, our study showed that there was no statistically significant difference between the two groups in terms of ICU length of stay, hospital length of stay, and incidence of new ICU organ dysfunction, and conservative oxygen therapy did not significantly improve the overall prognosis of critically ill patients. Considering that the condition of ICU patients is critical and complex and the prognosis and outcome of patients are affected by the severity of the disease and various treatment methods, a single conservative oxygen therapy strategy has limited impact on the prognosis of patients.

Limitations of this study are as follows: ① the target value of SpO_2_ set by conservative oxygen therapy is not uniform, and there is still a lack of high-quality evidence to define conservative oxygen therapy strategies. ② The included populations are different, such as severe pneumonia, septic shock, and ARDS. ③ The treatment levels of the included literature studies vary.

In conclusion, this meta-analysis included a large number of domestic and foreign literature studies and a large number of cases, and the heterogeneity of each literature is low. The analysis results show that compared with conventional oxygen therapy, the conservative oxygen therapy can reduce the short-term mortality rate of critically ill patients, as well as the incidence of ICU new inflections and incidence of ICUAW. Of course, a large number of high-quality RCT studies are still needed to be further confirmed in the future to provide more evidence-based medical evidence for clinical practice.

## Figures and Tables

**Table 1:** Basic characteristics of included studies.

Study	Year	Participants	Nr.		Intervention assignments		Outcome
			Conservative group	Conventional group	Conservative group	Conventional group	
Panwar et al. [16]	2016	Mechanically ventilated patients	52	51	SpO_2_ 0.88–0.92	SpO_2_ ≥ 96%	①②③④
Schjørring et al. [17]	2021	Patients with ARDS	1441	1447	PaO_2_ 60 mmHg	PaO_2_ 90 mmHg	②⑤
Asfar et al. [18]	2017	Patients with septic shock	217	217	SpO_2_ 0.88–0.95	FiO_2_ of 1.0 for 24 h	①②③⑤⑥⑦
Girardis et al. [10]	2016	ICU patients	216	218	SpO_2_ 0.94–0.98 or PaO_2_ 70–100 mmHg	PaO_2_ up to 150 mmHg or SpO_2_ 0.97–1.0	①③④⑤⑥
Mackle et al. [15]	2020	Mechanically ventilated patients	484	481	SpO_2_ 90–97%	No specific limiting FiO_2_ or SpO_2_	②
Yang and Wang [19]	2021	Mechanically ventilated patients	55	51	PaO_2_ 70∼100 mmHg or SpO_2_ 0.90∼0.92	PaO_2_ > 150 mmHg or SpO_2_ > 0.96	①③④⑤⑥⑦
Gelissen et al. [20]	2021	Critically ill patients with SIRS	205	195	PaO_2_ 60–90 mmHg	PaO_2_ 105–135 mmHg	①③⑤
Barrot et al. [14]	2020	Patients with ARDS	99	102	PaO_2_ 55–70 mmHg; SpO_2_ 88–92%	PaO_2_ 90–105 mmHg; SpO_2_ ≥ 96%	①②⑤⑥
Yang et al. [21]	2019	ICU patients	100	114	SpO_2_ 90–95%	SpO_2_ 96–100%	①
Martin et al. [22]	2021	Mechanically ventilated patients	17	17	SpO_2_ 88–92%	SpO_2_ 96%	①②

① Short-term mortality rate (28-day mortality rate or ICU mortality rate), ② 90-day mortality rate, ③ ICU length of stay, ④ hospital length of stay, ⑤ incidence of new ICU organ dysfunction, ⑥ incidence of new ICU infection, and ⑦ incidence of ICUAW.

## Data Availability

The data used to support the findings of this study are included within the article and the supplementary information files.

## Supplementary Materials

Supplementary Materials Supplementary materials included search strategies. Sup1: risk of bias graph: review of authors' judgements about each risk of bias item presented as percentages across all included studies. Sup2: meta-analysis of 90-day mortality in different groups. Sup3: meta-analysis of ICU length of stay in different groups. Sup4: meta-analysis of hospital length of stay in different groups. Sup5: meta-analysis of incidence of new ICU organ dysfunction in different groups. Sup6: meta-analysis of incidence of new ICU infections in different groups. Sup7: meta-analysis of incidence of ICUAW in different groups. Sup8: funnel plots of studies on short-term mortality.
