# Supplementary material for: Effects of Conservative Oxygen Therapy versus Conventional Oxygen Therapy on the Mortality in ICU Patients: A Meta-Analysis
Source: Can Respir J. 2023 Oct 14;2023:7023712. doi: 10.1155/2023/7023712 (PMC10590270; doi:10.1155/2023/7023712)

search strategies

((((((((Hypoxia[MeSH Terms]) OR (Hyperoxia[MeSH Terms])) OR (Oxygen Deficiencies[Title/Abstract])) OR (Hypoxemia[Title/Abstract])) OR (Anoxia[Title/Abstract])) OR (conservative oxygen[Title/Abstract])) OR (liberal oxygen[Title/Abstract])) OR (conventional oxygen[Title/Abstract])) AND (((("Critical Illness"[Mesh]) OR ("Critical Care"[Mesh])) OR ("Intensive Care Units"[Mesh])) OR (critically ill[Title/Abstract] OR acutely ill[Title/Abstract] OR intensive care[Title/Abstract] OR critical care[Title/Abstract] OR ICU[Title/Abstract] OR coronary care unit[Title/Abstract] OR neurological intermediate care unit[Title/Abstract])) Filters: Randomized Controlled Trial

Sup1 Risk of bias graph: review authors' judgements about each risk of bias item presented as percentages across all included studies.


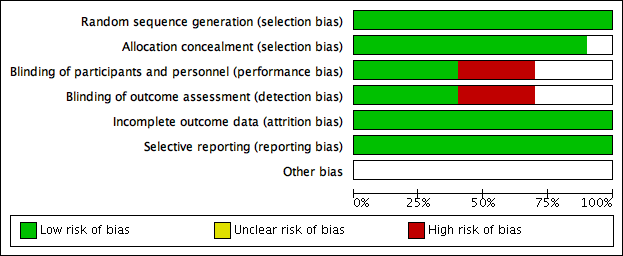


Sup2 Meta-analysis of 90-day mortality in different groups


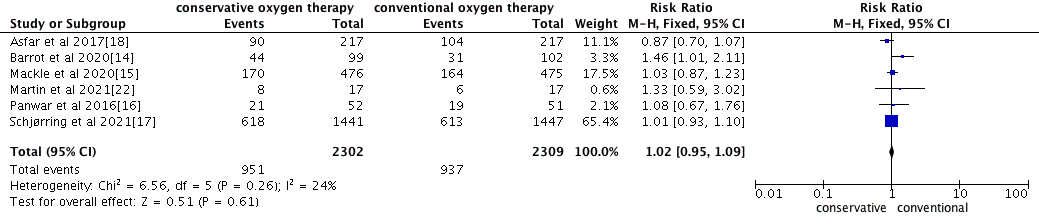


Sup3 Meta-analysis of ICU length of stay in different groups


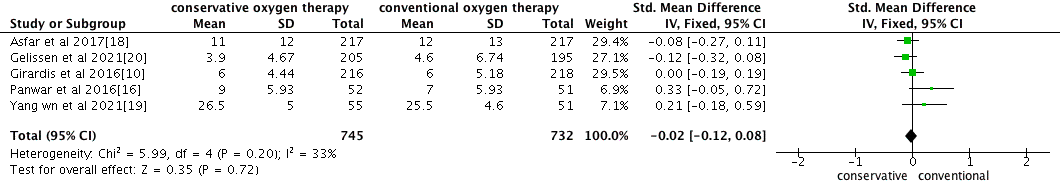


Sup4 Meta-analysis of hospital length of stay in different groups


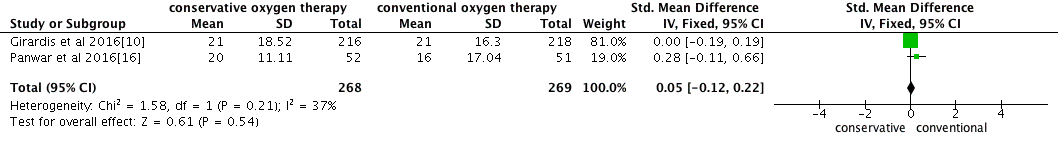


Sup5 Meta-analysis of incidence of new ICU organ dysfunction in different groups


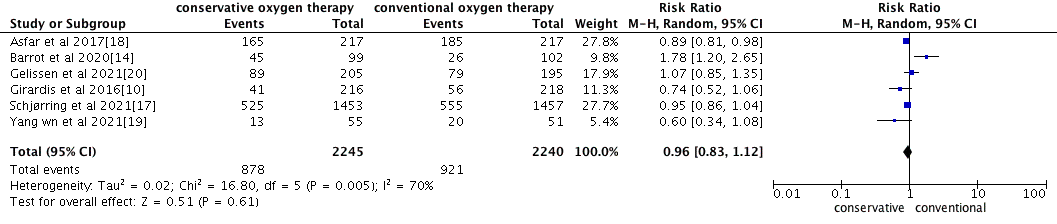


Sup6 Meta-analysis of incidence of new ICU infections in different groups


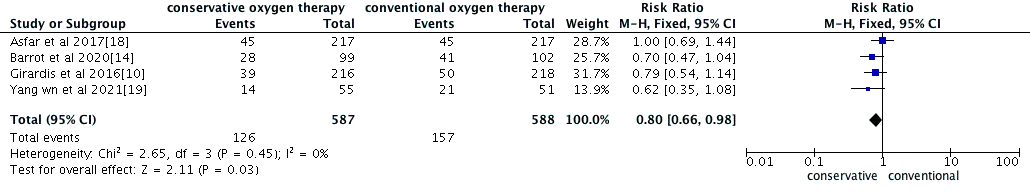


Sup7 Meta-analysis of incidence of ICUAW in different groups


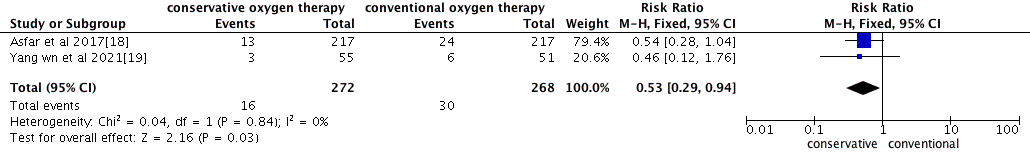


Sup8 Funnel plots of studies on short-term mortality


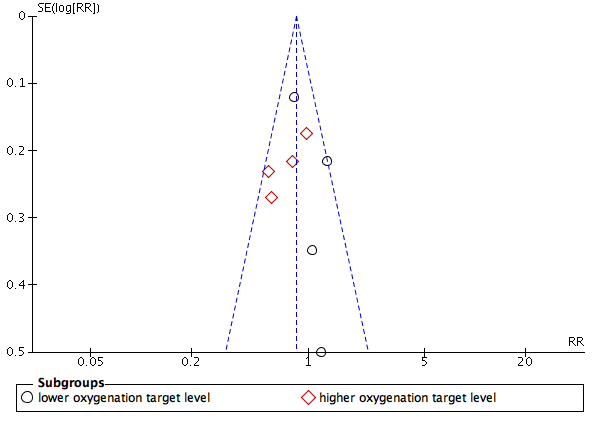

Supplement: Supplementary Materials — included search strategies. Sup1: risk of bias graph: review of authors' judgements about each risk of bias item presented as percentages across all included studies. Sup2: meta-analysis of 90-day mortality in different groups. Sup3: meta-analysis of ICU length of stay in different groups. Sup4: meta-analysis of hospital length of stay in different groups. Sup5: meta-analysis of incidence of new ICU organ dysfunction in different groups. Sup6: meta-analysis of incidence of new ICU infections in different groups. Sup7: meta-analysis of incidence of ICUAW in different groups. Sup8: funnel plots of studies on short-term mortality. [file 7023712.f1.docx]
